# Supplementary material for: MetaRibo-Seq measures translation in microbiomes
Source: Nat Commun. 2020 Jun 29;11:3268. doi: 10.1038/s41467-020-17081-z (PMC7324362; doi:10.1038/s41467-020-17081-z)
Supplement: Supplementary file 10 — Supplementary Data 7 [file 41467_2020_17081_MOESM10_ESM.zip › File2/Confidence_High_Taxonomy/57495_out.krona.html]

Javascript must be enabled to view this page.

members
magnitude
magnitudeUnassigned
count
unassigned
taxon
rank

57495\_out

68

68
superkingdom
2

976
phylum
68

68
200643
class

171549
order
68

171552
family
67

67
genus
838

1263102
species

SRS013687\_contig\_number\_27909SRS016541\_contig\_number\_16186SRS023914\_contig\_number\_21133
3

64

SRS011134\_contig\_number\_17823SRS012916\_contig\_number\_contig-100\_1012.100871SRS012969\_contig\_number\_9387SRS012989\_contig\_number\_6557SRS013098\_contig\_number\_21263SRS013521\_contig\_number\_9048SRS013638\_contig\_number\_2967SRS014415\_contig\_number\_1771SRS015590\_contig\_number\_7052SRS015782\_contig\_number\_25575SRS015794\_contig\_number\_5317SRS017307\_contig\_number\_12781SRS017375\_contig\_number\_12SRS017849\_contig\_number\_contig-100\_4334.4334SRS018888\_contig\_number\_264SRS019285\_contig\_number\_53SRS019808\_contig\_number\_13109SRS019910\_contig\_number\_18137SRS022609\_contig\_number\_42048SRS023526\_contig\_number\_317SRS024132\_contig\_number\_32962SRS043841\_contig\_number\_9030SRS044535\_contig\_number\_12820SRS045713\_contig\_number\_9364SRS046502\_contig\_number\_10741SRS047044\_contig\_number\_12339SRS047433\_contig\_number\_10898SRS049712\_contig\_number\_18309SRS049896\_contig\_number\_25058SRS049959\_contig\_number\_25752SRS050752\_contig\_number\_10514SRS053356\_contig\_number\_33908SRS053398\_contig\_number\_18379SRS058145\_contig\_number\_1334SRS064757\_contig\_number\_14608SRS065397\_contig\_number\_502SRS074586\_contig\_number\_2527SRS077194\_contig\_number\_8176SRS077641\_contig\_number\_553SRS077849\_contig\_number\_contig-100\_2506.176753SRS077991\_contig\_number\_12SRS078176\_contig\_number\_12947SRS078419\_contig\_number\_contig-100\_1128.183766SRS097958\_contig\_number\_6048SRS103987\_contig\_number\_278SRS104036\_contig\_number\_70SRS1041031\_contig\_number\_16920SRS1041116\_contig\_number\_3929SRS1041134\_contig\_number\_1048SRS1041137\_contig\_number\_23764SRS1041140\_contig\_number\_12205SRS104327\_contig\_number\_979SRS104912\_contig\_number\_15152SRS105082\_contig\_number\_618SRS143181\_contig\_number\_6181SRS143876\_contig\_number\_18264SRS144506\_contig\_number\_34664SRS144537\_contig\_number\_33145SRS144603\_contig\_number\_1615SRS144714\_contig\_number\_16277SRS146764\_contig\_number\_2911SRS147614\_contig\_number\_7871SRS148253\_contig\_number\_8627SRS150029\_contig\_number\_19705
165179
species

family
1853231
1

genus
283168
1

1
28118
species

SRS012849\_contig\_number\_14306
